# Serum Biomarkers of Renal Fibrosis: A Systematic Review

**DOI:** 10.3390/ijms232214139

**Published:** 2022-11-16

**Authors:** Alice Barinotti, Massimo Radin, Irene Cecchi, Silvia Grazietta Foddai, Elena Rubini, Dario Roccatello, Savino Sciascia

**Affiliations:** 1 University Center of Excellence on Nephrologic, Rheumatologic and Rare Diseases (ERK-Net, ERN-Reconnect and RITA-ERN Member) with Nephrology and Dialysis Unit and Center of Immuno-Rheumatology and Rare Diseases (CMID), Coordinating Center of the Interregional Network for Rare Diseases of Piedmont and Aosta Valley, San Giovanni Bosco Hub Hospital, 10154 Turin, Italy; 2 Department of Clinical and Biological Sciences, University of Turin, 10125 Turin, Italy

**Keywords:** chronic kidney disease, renal fibrosis, biomarkers

## Abstract

Chronic kidney disease (CKD) is a widely diffuse pathological condition which deeply impacts upon an affected patient’s quality of life and its worldwide rate is predicted to further rise. The main biological mechanism underlying CKD is renal fibrosis, a non-reversible process representing, for the affected system, a point of no return of tissue damage and dysfunction, deeply reducing the possible therapeutic strategies at the disposal of physicians. The best tool clinicians can use to address the extent of renal fibrosis at any level (glomeruli, tubule-interstitium, vasculature) is kidney biopsy that, despite its overall safety, remains an invasive procedure showing some shortcomings. Thus, the identification of novel non-invasive renal fibrosis biomarkers would be of fundamental importance. Here, when systematically reviewing the available evidence on serological biomarkers associated with renal fibrosis evaluated in patients suffering from CKD in the last five years, we found that despite the presence of several promising biomarkers, the level of observed evidence is still very scattered. Probably, the use of multiple measures capable of addressing different aspects involved in this condition would be the most suitable way to capture the high complexity characterizing the renal fibrotic process, having consequently a great impact on clinical practice by maximizing prevention, diagnosis, and management.

## 1. Introduction

Chronic kidney disease (CKD) is one of the most important diseases in terms of morbidity and mortality, and its progressive evolution deeply impacts on the quality of life of the affected patients. In fact, recent studies have shown that around 10% of the population is affected by CKD, and millions die every year because of the impossibility to access affordable treatment [1,2]. Despite the outstanding progress of modern medicine, the worldwide rate of CKD is predicted to further rise, given the aging of the population and the parallel increase in the prevalence of numerous diseases leading to the condition [3,4,5,6]. As reported in a recent work by the Global Burden Disease (GBD) Chronic Kidney Disease Collaboration, the prevalence of CKD is estimated as 9.1% in the general population, with CKD stages 1–2 accounting for 5%, stage 3 for 3.9%, stage 4 for 0.16%, and stage 5 for 0.07% [7]. In most cases, the outcome is nefarious and patients inevitably undergo end-stage renal disease (ESRD), dialysis, or kidney transplantation. The estimated number of patients receiving renal replacement therapy accounts for more than 2.5 million, a number that is projected to double by 2030 [7,8]. Moreover, this number may only represent 10% of people who need treatment to live [8], and the situation is even more severe if we consider that CKD represents an independent risk factor for cardiovascular disease (CVD), impacting also upon CVD mortality [7,9,10]. Thus, CKD represents a global health burden, with significant costs required to manage the clinical complexity of these patients.

While a growing body of evidence is supporting researchers worldwide to understand the magnitude of the problem, we are currently only scratching the surface of the complex systems of interactions leading to CKD. The main biological mechanisms characterizing CKD, slowly leading to kidney failure and dysfunction, are represented by renal fibrosis.

Fibrosis is a non-reversible process that represents, for the affected system, a point of no return of tissue damage and dysfunction, deeply reducing the possible therapeutic strategies at the disposal of clinicians [11,12].

The fibrotic process could be described as a “failed wound healing”, or as an excessive accumulation of extracellular matrix (ECM). Allowing tissue regeneration, which is a fundamental response to injury, is important, but the exaggeration of this event leads to a pathologic outcome [13,14]. Chronic fibrogenesis induces a shift from supportive fibrotic tissue to a microenvironment in which the increase in the number and activity of ECM-producing cells results in excessive ECM deposition, and consequently in the disruption of the normal parenchymal architecture, interfering with organ function [15,16,17].

Measurements such as serum creatinine, estimated glomerular filtration rate (eGFR), and albuminuria are routinely used when evaluating CKD patients, but they predominantly reflect glomeruli health, and they cannot fully capture all the components of kidney damage. The best tool that clinicians can use to this purpose is kidney biopsy, which allows them to evaluate the severity of tissue damage at any level: glomeruli, tubule-interstitium, and vasculature [18,19,20]. Indeed, together with glomerulosclerosis, tubulointerstitial fibrosis and atherosclerosis are common findings in most CKD forms and their severity results are reliable to predict kidney failure progression. Nonetheless, regardless of its overall safety, biopsy remains an invasive procedure [18,21,22] and its use to periodically monitor the progression of the diseases is limited to specific circumstances.

The identification of biomarkers that allow for the estimation of kidney fibrosis and damage progression in a non-invasive manner would be of fundamental importance, in order to help treating physicians to tailor strategies to improve patients’ diagnosis, management, and responsiveness to therapy. Nowadays however, evidence on new biomarkers and their association to the histological findings are still heterogeneous and a consensus is still lacking.

The aim of this systematic review was to evaluate the recent evidence supporting the use of available serologic biomarkers to investigate the degree of kidney fibrosis on kidney biopsy.

## 2. Methods

A detailed literature search has been developed *a priori* to identify articles that reported findings from clinical and laboratory studies that evaluated the prognostic or diagnostic role of serological biomarkers in pathologic conditions characterized by renal fibrosis. Keywords and subject terms included: ((“renal fibrosis”[MeSH Terms]) OR (“renal”[All Fields] AND “fibrosis”[All Fields]) OR (“renal fibrosis”[All Fields]) OR (“kidney fibrosis”[MeSH terms]) OR (“kidney”[All Fields] AND “fibrosis”[All fields]) OR (“chronic kidney disease”[MeSH terms])) AND ((“serum biomarkers”[MeSH Terms]) OR (“biomarkers”[All Fields])). The search strategy was applied to Ovid MEDLINE, In-Process, and Other Non-Indexed Citation and Ovid Medline for the last five years (from May 2017 to May 2022).

**Figure 1:**
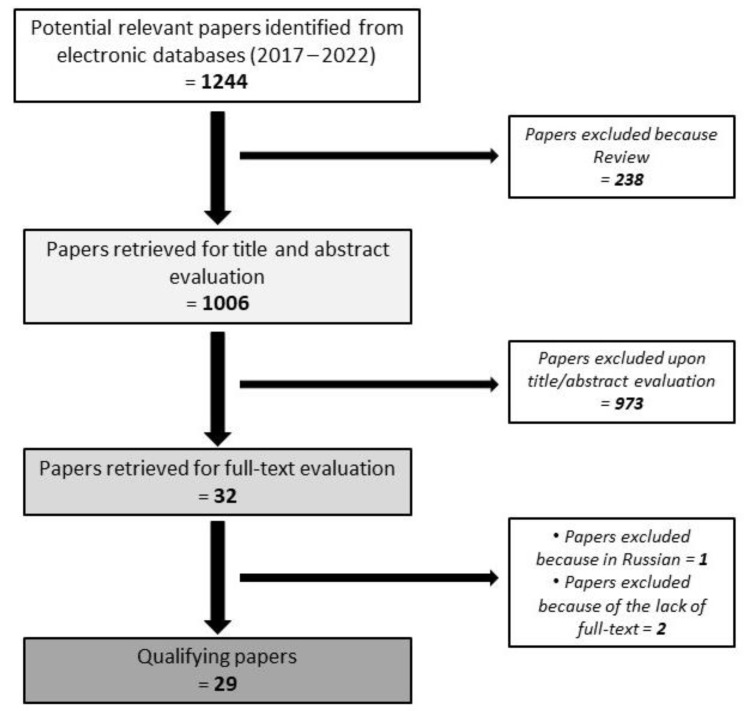
Literature search strategy and studies selection.

The studies identified were systematically analyzed by two independent reviewers (AB and MR). Disagreements were resolved by consensus; if consensus could not be achieved, a third party (SS) would provide an assessment of eligibility. As the data on eligibility were dichotomous (eligible: yes/no), agreement at both the title and abstract review and the full article review stages was determined by calculation of Cohen’s kappa coefficient (k = 0.92). Literature search strategy is shown in Figure 1.

Inclusion criteria of the studies were as follow:

- inclusion of at least 25 patients;
- publication in the last five years;
- presentation of clinical related data (not just in vitro analysis).

## 3. Results and Discussion

The literature search identified 29 studies that were eligible according to the inclusion criteria and they have been included in the systematic review [23,24,25,26,27,28,29,30,31,32,33,34,35,36,37,38,39,40,41,42,43,44,45,46,47,48,49,50,51]. A detailed literature search strategy is displayed in Figure 1. Table 1 resumes the main characteristics of the included studies.

When focusing on the study design, 2 out of 29 (6.9%) were post-hoc analyses, 8/29 (27.6%) were prospective, and 19/29 (65.5%) were retrospective studies.

When considering the population included in the selected studies, a total of 8889 patients were recruited. Details regarding the diagnosis of these patients are reported in Table 2.

Moreover, 17 studies out of 29 (58.6%) also included additional groups of subjects as controls. Most of them were healthy donors (684), while other studies included controls as subjects with renal diseases (Table 1).

A total of 65 different biomarkers were evaluated in patients’ serum to assess renal fibrosis and a statistically significant association has been found between 42 of them and/or other fibrotic biomarkers/histopathological findings (Table 1). Among these, the most frequently tested were: MCP-1 (monocyte chemoattractant protein-1) (4 studies out of 29 = 13.8%), KIM-1 (kidney injury molecule-1) (3/29 = 10.3%), MMP-7 (matrix metalloproteinase 7) (3/29 = 10.3%), Pro-C3 (pro-peptide of type III collagen) (3/29 = 10.3%), Pro-C6 (pro-peptide of type VI collagen)(3/29 = 10.3%), TNFR-1 (tumor necrosis factor receptor 1) (3/29 = 10.3%), and TNFR-2 (tumor necrosis factor receptor 2) (3/29 = 10.3%). The main findings are briefly discussed separately.

### 3.1. MCP-1 (Monocyte Chemoattractant Protein-1) and KIM-1 (Kidney Injury Molecule-1)

MCP-1 has been tested in 4 studies out of 29 (13.8%), while KIM-1 has been in tested in 3 out of 29 (10.3%). MCP-1 belongs to the C-C chemokine family. It is produced by many cell types, but it is mainly expressed by activated monocytes/macrophages, T cells, and natural killer cells, and plays a role in leukocyte infiltration to the kidney [52,53]. KIM-1 is a transmembrane protein of proximal tubule cells whose expression results to be strongly upregulated during tubule damage [54,55]. These two biomarkers are indeed used to evaluate tubule injury when facing patients affected by renal pathological conditions. In particular, MCP-1 seems to be useful to assess the extent of inflammation and fibrotic activity, while KIM-1 seems reliable for quantifying the severity of tubule cell injury [19,56].

### 3.2. MMP-7 (Matrix Metalloproteinase 7)

MMP-7, or matrilysin, has been tested in 3 studies out of 29 (10.3%). It is a secreted zinc- and calcium-dependent endopeptidase able to degrade different substrates, both of the extracellular matrix and the basement membrane [57,58]. It is indeed a downstream target gene of Wnt/β-catenin signaling and growing evidence indicates that MMP-7 seems to play an important role in the pathogenesis of kidney fibrosis and it is a useful biomarker to predict kidney disease progression [59,60].

### 3.3. Pro-C3 (Pro-Peptide of Type III Collagen)

Pro-C3 has been tested in 3 studies out of 29 (10.3%). Pro-C3, represents the N-terminal pro-peptide of type III procollagen that detects the formation of type III collagen which, together with collagen type I, constitutes the major component of the ECM. Excessive ECM deposition is a hallmark of fibrosis and collagen type III seems to be dominant in the early stages of this pathological process. As a result, Pro-C3 has gained interest in the last years when assessing fibrogenesis [40,61].

### 3.4. Pro-C6 (Pro-Peptide of Type VI Collagen)

Pro-C6 has been tested in 3 studies out of 29 (10.3%). It is produced by fibroblasts and is found at the interface between the interstitial matrix and the glomerular basement membrane in the kidney [62]. Pro-C6 has been observed to be expressed at low levels in healthy individuals and overexpressed in patients with renal fibrosis. Interistingly, its released fragment (endothropin-ETP) has been demonstrated to increase TGF-β expression, to promote EMT, chemotaxis of macrophages, adipose tissue fibrosis and metabolic dysfunction [63]. 

### 3.5. TNFR-1 (Tumor Necrosis Factor Receptor 1) and TNFR-2 (Tumor Necrosis Factor Receptor 2)

TNFR-1 and TNFR-2 have both been tested in 3 studies out of 29 (10.3%). It has been observed that by binding their ligand TNF-α, they play a role in the kidney fibrotic process. TNF-α is indeed a potent mediator of the inflammatory response produced by various cell types, including macrophages, mesangial cells, and tubular epithelial cells [64,65]. High serum levels of TNF-α have been observed in human CKD and experimental kidney disease models, and they also positively correlated with the severity of kidney injury. Besides this, in unilateral ureteral obstruction models, the inhibition of TNFR-1 has been associated with an anti-fibrotic response [66,67].

### 3.6. Serum Biomakers and Their Relationship with Histologic Findings at Kidney Biopsy

When analyzing the included studies more in depth, it was possible to further divide them in two sub-groups based on the availability of the comparison between serologic biomarkers and histologic findings. In detail, the first group comprises those studies in which a comparison between circulating biomarkers and the findings from kidney biopsy has been performed (14 studies out of 29 = 48.3%).

Moreover, in 8 out of 14 studies (57.1%) the grading of fibrosis was specified, while 6/14 (42.9%) did not apply any scoring system or grading of kidney fibrosis. Table 3 summarizes the studies that included kidney biopsy in the analysis and the scores used to assess the grade of fibrosis. The second group did not compare the biomarkers to histological features (15 studies out of 29 = 51.7%), but to other biomarkers that are presumed to have a role in CKD and fibrosis according to the literature.

When focusing on the first group, we observed that the following serum biomarkers were associated with fibrosis: MMP-7 (matrix metalloproteinase 7), C3M (MMP-mediated degradation of collagen type III), Pro-C6 (pro-peptide of type VI collagen), Klotho, HE4 (human epididymis secretory protein 4), miR-181 (microRNA-181), PTX-2 (pentraxin-2), LOX (lysyl oxidase), CDH11 (cadherin-11), SMOC2 (sparc-related modular calcium binding protein-2), PEDF (pigment epithelium-derived factor), RelB (transcription factor RelB), ETP (endotrophin), NGAL (neutrophil gelatinase-associated lipocalin), cystatin C, TGF-β1 (transforming growth factor β1), TGF-β2 (transforming growth factor β2), TGF-β3 (transforming growth factor β3), BMP-7 (bone morphogenetic protein 7), uromodulin, and DKK-3 (dickkopf-related protein 3). Two of them have been used in more than one study, indeed in two studies (Pro-C6 and HE4), while the others have been used in only one work. Interestingly, among those biomarkers that were found to be associated with fibrosis in the overall cohort of patients included in the present systematic review, only MMP-7 maintained this association with biopsy-proven renal fibrosis.

Renal fibrosis represents the main pathological process leading to CKD. The scarring can affect every compartment of the kidney structure, separately or in concomitance, resulting in glomerulosclerosis, tubule-interstitial fibrosis, atherosclerosis causing parenchymal damage, ultimately leading to kidney dysfunction [11,12]. Fibrosis is a complex and dynamic process, in which the crosstalk between different cell types is crucial along with multiple cellular and molecular cascade. After an initial injury, the kidney attempts to repair the damage by activating the resident cells, inducing the production of pro-inflammatory cytokines and chemokines. Consequently, infiltration of inflammatory monocytes/macrophages and T-cells is promoted, which in turn stimulate mesangial cells, fibroblasts and tubular epithelial cells to undergo a phenotypic activation or transition, leading to the production of ECM components [11,12].

A renal fibrosis targeted therapy is still in its beginnings, nevertheless this growing knowledge at cellular and molecular level holds considerable hope for the future and a few points are worth to be considered. Direct or indirect quantitative assessment of the degree of kidney fibrosis damage and progression would be extremely important to identify patients more prone to undergo a worsening of their condition and also to identify safe and effective treatments for renal fibrosis in the course of various type of CKD.

In this setting, biomarkers of fibrosis have gained an increasing importance when facing CKD, from a diagnostic to a therapeutic point of view. The relative lack of non-invasive surrogate outcome measures that specifically assess renal fibrosis in general represents one of the major barriers to clinical interventional trials. The currently used non-invasive parameters such as the eGFR, on one hand lack to fully capture all the components of kidney damage and in particular tubule damage, which has been observed to be the most reliable aspect for predicting kidney failure progression [18,19], and on the other hand, their utility can be limited by the sluggish evolution of some renal diseases. Kidney biopsy thus, still represents the gold standard for this purpose. However, it is an invasive, although overall safe, procedure that can be repeated only a limited number of times [21,22]. 

In this systematic review, we analyzed the available evidence on serological biomarkers associated with renal fibrosis in patients suffering from renal diseases. A study with a similar purpose has been carried out in 2017 by Mansour and colleagues, where an association between renal fibrosis and MMP-2, MCP-1 and TGF-β was reported. When analyzing the 29 included studies, in which a total of 65 biomarkers have been tested, 42 potential biomarkers were showed to have a significant positive association with fibrosis in the analysed patients. Not surprisingly, the most frequently tested molecules were known circulating biomarkers associated to the inflammatory process (e.g., MCP-1, TNFR-1 and TNFR-2), kidney damage, (e.g. KIM-1), and extracellular matrix remodeling (e.g., MMP-7, Pro-C3 and Pro-C6). However, most of these studies did not confirm their findings histologically. When focusing specifically on the sub-group of studies in which a direct comparison of serologic and histologic findings was performed, the results were very heterogenous and the only analytes that were confirmed to be significantly associated with fibrosis were MMP-7 and Pro-C6. 

Some limitations must be acknowledged. First, this systematic review included data derived from studies with different design and methods, therefore limiting direct comparability. Second, the population evaluated was highly heterogeneous, showing patients affected by diverse kidney-related diseases. Further, most of the studies did not compare their results to histological findings. Nonetheless, it should also be considered that the scope of this work was to investigate the serum biomarkers most used in the last years to evaluate renal fibrosis. We acknowledge also that some arbitrary choices have been performed when implementing our a priori research strategy, such as including only literature from the last five years or limiting our analysis to studies with more than 25 patients. However, these implementations were applied to improve the novelty and comparability of the results.

## 4. Conclusions

To date, evidence regarding potential novel biomarkers to assess kidney fibrosis is still very scattered and none of the biomarkers are routinely employed in clinical practice. Thus, further studies are needed. Nonetheless, this systematic review highlighted how the idea of a panel combining different biomarkers, rather than the employment of a single one, could be the best path to follow. Multiple measures capable of addressing the different aspects involved in this pathological condition, such as glomerular and tubular injury and dysfunction, and inflammation could be the most suitable way to capture the high complexity characterizing renal fibrotic process, having consequently a great impact on clinical practice by maximizing prevention, diagnosis, and management.

In the near future, more specialized tools (ranging from molecular imaging to laboratory testing) will be integrated into clinical practice to assess the ongoing pathological processes within the kidneys ultimately improving disease staging and prognosis, monitoring treatment responses. Such developments will change how clinicians treat and manage patients suffering from renal fibrosis and will bring nephrology precision medicine closer.

## Figures and Tables

**Table 1:** Summary of the main characteristics of the studies included in the systematic review.

Ref	Authors	Year	Design	N Patients	Patients Population	Controls	Tested Biomarkers	Biomarkers Statistically Associated with Fibrosis
[23]	Zhang et al.	2017	R	244	IgA nephropathy	40 HC	MMP-7	MMP-7
[24]	Stribos et al.	2017	R	78	Renal transplant recipients	NA	C3M, Pro-C3, C4M, C5M, Pro-C6, C6M	C3M, Pro-C6
[25]	Akin et al.	2017	PR	81	AKI (44), CKD (37)	NA	HA	HA
[26]	Chen et al.	2017	R	31	CKD	25 HC	Bcl-3	Bcl-3
[27]	Cho et al.	2018	R	67	IgA nephropathy (26), FSGS (12), MCD (7), MN (3), TBMD (3), MPGN (2), post-infectious glomerulonephritis (1), LN (1), GN (1), ATN (1), amyloidosis (1), non-specific findings (9)	NA	Klotho	Klotho
[28]	Luo et al.	2018	R	103	Renal transplant recipients	127 HC	HE4	HE4
[29]	Nielsen et al.	2018	PR	492	CKD	NA	LAMC1	LAMC1
[30]	Yiang et al.	2018	post hoc	230	CKD	67 HC	Bmi-1	Bmi-1
[31]	Zhang et al.	2019	R	58	Biopsy-proven renal fibrosis	10 HC	miR-181	miR-181
[32]	Ren et al.	2019	R	697	DN	150 HC	VASH-1, SIRT1, HIF1α, VEGF, CRP, TNF-α, TGF-β1	VASH-1
[33]	Ozkan et al.	2019	R	131	CKD	34 HC	PCPE-1	PCPE-1
[34]	Basturk et al.	2020	R	45	CKD	16 HC	PTX-2	Pentraxin-2 (PTX-2)
[35]	Bieniaś et al.	2020	R	45	Unilateral hydronephrosis (children)	21 HC	MMP-1, MMP-2, MMP-9, TIMP-1 and TIMP-2	MMP-1, MMP-2, MMP-9, TIMP-1 and TIMP-2
[36]	Ihara et al.	2020	PR	1181	Type II diabetes	NA	WFDC2, MMP-7	WFDC2 and MMP-7
[37]	Zhang et al.	2020	R	202	IgA nephropathy (43), MN (42), DN (28), hypertensive nephrosclerosis (21), MCD (16), ANCA-associated nephritis (12), minor histopathology abnormality (11), LN (8), FSGS (5), renal amyloidosis (5), cast nephropathy (5), ORG (2), TMA (2), ATN (1), uric acid nephropathy (1)	30 HC	LOX	LOX
[38]	Musiał et al.	2020	R	70	Children with CKD: obstructive uropathy (23), hypo-/dysplastic kidneys (15), reflux nephropathy (14), PKD (4), other genetic disorders (5), AKI (4), and unknown factors (5)	12 children with monosymptomatic nocturnal enuresis and normal kidney function	MCP-1, MCSF, TIMP-2, BIRC5	MCP-1, MCSF, TIMP-2, BIRC5
[39]	Schrauben et al.	2020	PR	894	DN	NA	KIM-1, TNFR-1, TNFR-2, MCP-1, suPAR, YKL-40	KIM-1, TNFR-1, TNFR-2, MCP-1, suPAR, YKL-40
[40]	Genovese et al.	2020	PR	500	CKD	NA	Pro-C3, C3M	Pro-C3, C3M
[41]	Jie et al.	2021	R	168	CKD	NA	UMOD	UMOD
[42]	Schmidt et al.	2021	R	973	CKD	snRNA-seq dataset derived from 3 healthy kidneys	CDH11, SMOC2, PEDF, MGP, TSP-2	CDH11, SMOC2, and PEDF
[43]	Sun et al.	2021	R	47	CKD	60 HC	RelB, HE4	RelB, HE4
[44]	Sparding et al.	2021	R	96	IgA nephropathy (49), ANCA-associated vasculitis (47)	85 IgAN (validation cohort), 10 HC	ETP (Pro-C6)	ETP (Pro-C6)
[45]	Gutiérrez et al.	2021	PR	594	Type II diabetes	NA	TNFR1, TNFR2, suPAR, MCP-1, YKL-40, KIM-1	TNFR1, TNFR2, YKL-40
[46]	Liu et al.	2021	post hoc	231	Type II diabetes and stage 3 CKD	NA	PDGF-AA, PDGF-BB, MCD, FGF2, GMCSF, INFα2, MCP-3, IL-12p70, sCD40L, IL-2, IL-6, IL-8, MIP-1α, NGAL, cystatin C	
[47]	Genovese et al.	2021	R	40	LN	SLE without LN (20), HC (20), biopsy-proven histologic kidney inflammation/damage without SLE (10)	Pro-C3, Pro-C6	Pro-C6
[48]	Naicker et al.	2021	R	25	HIV-positive CKD	25 HIV-positive without CKD, 24 HC	NGAL, cystatin C, TGF-β1, TGF-β2, TGF-β3, BMP-7	NGAL, cystatin C, TGF-β1, TGF-β2, TGF-β3, BMP-7
[49]	Enoksen et al.	2021	PR	1302	NA, general population	NA	MMP-2, MMP-7, TIMP1	MMP-7
[50]	Chan et al.	2022	R	132	Renal transplant recipients	NA	UMOD	UMOD
[51]	Sciascia et al.	2022	PR	132	75 SLE, 57 SLE with LN	50 HC	DKK-3	DKK-3

(MMP-7 = matrix metalloproteinase 7; C3M = MMP-mediated degradation of collagen type III; Pro-C3 = pro-peptide of type III collagen; C4M = MMP-mediated degradation of collagen type IV; C5M = MMP-mediated degradation of collagen type V; Pro-C6 = pro-peptide of type VI collagen; C6M = MMP-mediated degradation of collagen type VI; HA = hyaluronic acid; Bcl-3 = B cell lymphoma 3; WISP-1 = WNT1-inducible signaling pathway protein-1; HE4 = human epididymis secretory protein 4; LAMC1 = laminin subunit gamma 1; Bmi-1 = polycomb complex protein BMI-1; miR-181 = microRNA-181; VASH-1 = vasohibin-1; SIRT1 = sirtuin 1; HIF1α = hypoxia-inducible factor 1α; VEGF = vascular endothelial growth factor; CRP = C-reactive Protein; TNF-α = tumor necrosis factor α; TGF-β1 = transforming growth factor β1; PCPE-1 = procollagen C-proteinase enhancer-1; PTX-2 = pentraxin-2; MMP-1 = matrix metalloproteinase 1; MMP-2 = matrix metalloproteinase 2; MMP-9 = matrix metalloproteinase 9; TIMP-1 = tissue inhibitor of matrix metallopeptidase 1; TIMP-2 = tissue inhibitor of matrix metallopeptidase 2; WFDC2 = WAP four-disulfide core domain protein 2; LOX = lysyl oxidase; MCP-1 = monocyte chemoattractant protein-1; MCSF = macrophage colony-stimulating factor; BIRC5 = surviving; KIM-1 = kidney injury molecule-1; TNF-R1 = tumor necrosis factor receptor 1; TNF-R2 = tumor necrosis factor receptor 2; suPAR = soluble urokinase plasminogen activator receptor; YKL-40 = chitinase 3-like 1; UMOD = uromodulin; CDH11 = cadherin-11; SMOC2 = sparc-related modular calcium binding protein-2; PEDF = pigment epithelium-derived factor; MGP = matrix-Gla protein; TSP2 = thrombospondin-2; RelB = transcription factor RelB; ETP = endotrophin; PDGF-AA = platelet-derived growth factor AA; PDGF-BB = platelet-derived growth factor BB; FGF-2 = fibroblast growth factor 2; MDC = macrophage-derived chemokine; GMCSF = granulocyte-macrophage colony-stimulating factor; IFNα2 = interferon α2; MCP-3 = monocyte chemoattractant protein-3; IL-12p70 = interleukin 12p70; sCD40L = soluble cluster of differentiation 40-ligand; IL-2 = interleukin 2; IL-6 = interleukin 6; IL-8 = interleukin 8; MIP-1α = macrophage inflammatory protein 1α; NGAL = neutrophil gelatinase-associated lipocalin; TGF-β2 = transforming growth factor β2; TGF-β3 = transforming growth factor β3; BMP-7 = bone morphogenetic protein 7; DKK-3 = dickkopf-related protein 3; AKI = acute kidney injury; CKD = chronic kidney disease; FSGF = focal segmental glomerulosclerosis; DN = diabetic nephropathy; MCD = minimal change disease; MN = membranous nephropathy; TBMD = thin basement membrane disease; MPGN = membranous proliferative glomerulonephritis; LN = lupus nephritis; GN = crescentic glomerulonephritis; ATN = acute tubular necrosis; ORG = obesity-related glomerulopathy; TMA = thrombotic microangiopathy; PKD = polycystic kidney disease; NA = not applicable).

**Table 2:** Summary of patients’ diagnosis regarding the whole cohort included in the study.

Diagnosis	Patients Number (%)
Diabetic nephropathy	3625 (40.7)
CKD without specifying the underlying cause	2675 (30.1)
IgA nephropathy	362 (4.1)
Renal transplant recipients	313 (3.5)
Lupus nephritis	106 (1.2)
Systemic lupus erythematosus	75 (0.8)
Generically reported as biopsy-proven renal fibrosis	58 (0.6)
Acute kidney injury	50 (0.5)
ANCA-associated vasculitis	47 (0.5)
Unilateral hydronephrosis	45 children (0.5)
Membranous nephropathy	45 (0.5)
HIV-positive CKD	25 (0.3)
Minimal change disease	23 (0.25)
Obstructive uropathy	23 (0.25)
Hypertensive nephrosclerosis	21 (0.2)
Focal segmental glomerulosclerosis	17 (0.2)
Hypo/dysplastic kidney	15 (0.2)
Reflux nephropathy	14 (0.15)
ANCA-associated nephritis	12 (0.1)
Minor histopathologic abnormality	11 (0.1)
Renal amyloidosis	6 (0.05)
Cast nephropathy	5 (0.05)
Polycystic kidney disease	4 (0.05)
Thin basement membrane disease	3 (0.03)
Thrombotic microangiopathy	2 (0.02)
Membranous proliferative glomerulonephritis	2 (0.02)
Post-infectious glomerulonephritis	1 (0.01)
Crescentic glomerulonephritis	1 (0.01)
Uric acid nephropathy	1 (0.01)
General population (prospectively followed)	1302 (14.6)

**Table 3:** Summary of the studies that included kidney biopsy in their analysis and their scores to assess the grade of fibrosis.

Ref	Author	Year	N Patients	Biomarkers Tested	Biomarkers Statistically Associated with Fibrosis	Fibrosis Grade Assessment
[23]	Zhang et al.	2017	244	MMP-7	MMP-7	MEST-C (Oxford classification) [68]
[24]	Stribos et al.	2017	78	C3M, Pro-C3, C4M, C5M, Pro-C6, C6M	C3M, Pro-C6	Not specified
[27]	Cho et al.	2020	67	Klotho	Klotho	% of glomeruli affected by segmental or global glomerulosclerosis assessed by dividing the number of sclerosing glomeruli with the total number of acquired glomeruli; Degree of foot process effacement assessed based on the % of evaluated areas: absent, 0–10%; focal mild, 10–30%; focal moderate, 30–50%; focal severe, 50–70%; and diffuse, ≥70%; IFTA evaluated semi-quantitatively according to the proportion of the cortical area involved: absent <1%; mild 1–25%; moderate 25–50%; severe ≥50% of the total area; Intimal thickening assessed as the % of narrowed lumen of most severely affected blood vessel: absent 0%; mild <25%; moderate 25–50%; severe ≥50%.
[28]	Luo et al.	2018	103	HE4	HE4	Banff classification [69]
[31]	Zhang et al.	2019	58	miR-181	miR-181	Not specified
[34]	Basturk et al.	2020	45	PTX-2	PTX-2	Not specified
[37]	Zhang et al.	2020	202	LOX	LOX	Not specified
[42]	Schmidt et al.	2021	973	CDH11, SMOC2, PEDF, MGP, TSP2	CDH11, SMOC2, PEDF	IFTA was graded as involvement of <10%, 11–25%, 26–50%, or >50% of total cortical volume.
[43]	Sun et al.	2021	47	RelB, HE4	RelB, HE4	Not specified
[44]	Sparding et al.	2021	96	ETP	ETP	MEST-C (Oxford classification) [68], Banff classification [69]
[47]	Genovese et al.	2021	40	Pro-C3, Pro-C6	Pro-C6	% of interstitial fibrosis; % of tubular atrophy; Interstitial mononuclear cell infiltration.
[48]	Naicker et al.	2021	25	NGAL, cystatin C, TGF-β1, TGF-β2, TGF-β3, BMP-7	NGAL, cystatin C, TGF-β1, TGF-β2, TGF-β3, BMP-7	Not specified
[50]	Chan et al.	2022	132	Uromodulin	Uromodulin	Areas with fibrosis were determined at 5% level for each visual field and 1% level for averaged values. There was a high degree of concordance in IF% scores between the investigators, with intra- and inter-observer variability <5% in all but three cases.
[51]	Sciascia et al.	2022	132	DKK-3	DKK-3	ISN/RPS

(MMP-7 = matrix metalloproteinase 7; C3M = MMP-mediated degradation of collagen type III; Pro-C3 = pro-peptide of type III collagen; C4M = MMP-mediated degradation of collagen type IV; C5M = MMP-mediated degradation of collagen type V; Pro-C6 = pro-peptide of type VI collagen; C6M = MMP-mediated degradation of collagen type VI; HE4 = human epididymis secretory protein 4; miR-181 = microRNA-181; PTX-2 = pentraxin-2; LOX = lysyl oxidase; UMOD = uromodulin; CDH11 = cadherin-11; SMOC2 = sparc-related modular calcium binding protein-2; PEDF = pigment epithelium-derived factor; MGP = matrix-Gla protein; TSP2 = thrombospondin-2; RelB = transcription factor RelB; ETP = endotrophin; NGAL = neutrophil gelatinase-associated lipocalin; TGF-β2 = transforming growth factor β2; TGF-β3 = transforming growth factor β3; BMP-7 = bone morphogenetic protein 7; DKK-3 = dickkopf-related protein 3; Not specified = did not report any score or information regarding the way used to assess the grading of fibrosis).

## Data Availability

Data can be shared upon reasonable request to the corresponding author.
